# Deposition of Uniform Nanoscale Patterns on Silicon Dioxide Based on Coaxial Jet Direct Writing

**DOI:** 10.3390/polym15183702

**Published:** 2023-09-08

**Authors:** Shiwei Shi, Zeshan Abbas, Xiangyu Zhao, Junsheng Liang, Dazhi Wang

**Affiliations:** Key Laboratory for Micro/Nano Technology and System of Liaoning Province, Dalian 116024, China; hopenotout1214@mail.dlut.edu.cn (Z.A.); zxy4195@mail.dlut.edu.cn (X.Z.); jsliang@dlut.edu.cn (J.L.); d.wang@dlut.edu.cn (D.W.)

**Keywords:** CE-jet morphology, phase field model, drag, multiphysics, silicon dioxide, high-viscosity solution

## Abstract

To increase the printing stability of low-viscosity solutions, an auxiliary method was proposed using a coaxial electrohydrodynamic jet. A high-viscosity solution was employed as the outer layer in the printing process, and it could be removed (dissolved away) after printing the structures. A combination of mechanical and electrical forces was proposed to enhance the consistency, durability, and alignment of the printed versatile structures. The instability of the jet trajectory (which arose from the repulsion between the jet and the base with a residual charge, in addition to the winding effect of the solution) was also reduced using the drag force along the direction of movement. Moreover, the jet velocity, the surface charge, and the influence of various working voltages on the jet speed were simulated. An array of IDT-BT nanostructures measuring about 100 nm was prepared on silicon dioxide (using an inner needle with a diameter of 130 µm) by equating the moving speed (350 mm/s) of the substrate to the speed of the jet. Moreover, the moving speed (350 mm/s) of the substrate was compared exclusively to the speed of the jet. The method proposed throughout this study can provide a reference for enhancing the stability of low-viscosity solutions on substrates for high-efficiency fabrication devices (NEMS/MEMS).

## 1. Introduction

At present, electrohydrodynamic (EHD) jet printing is considered a very promising and reliable technique for alternative fabrication applications of high-resolution micro/nanostructures. The EHD printing technique is characterized by straightforward operation without contact or the employment of any molds or photo-masks for the preparation of nanoscale array structures and suspended structures. It is compared with conventional fabrication approaches such as counting lithography [1], laser ablation [2], and nanoimprint lithography [3] as well as mask printing and is considered an advantageous method [4]. EHD printing is an exceedingly efficient additive manufacturing process for the preparation of direct writing nanoscale structures [5]. Moreover, EHD printing technology is combined with nanoscale materials for their excellent printing properties and is widely used in nanowire sensors [5], field-effect transistors [6], micro/nanofiber-shaped optical polarizers [7], light emitters [8], energy harvesters [9], stretchable sensors [10], three-dimensional stacked nanoarchitectures [11], and tissue engineering [12]. The sensors and other microelectronic technologies are used to accommodate the technological requirements of the aforementioned aligned printed structures. A concise method should be adopted to determine the uniformity and order of the microstructures [13,14]. Since, the concept of coaxial jets was first put forward by Loscertales et al. [15], the E-jet technology advanced to the coaxial electrohydrodynamic jet (CE-jet). The coaxial needle consists of inner and outer capillaries, and the solutions are delivered through two syringe pumps. This technology is widely used in fabricating core–shell structures with the advantages of high packing precision, simple processes, and strong controllability [16,17].

However, the EHD printing process is easily affected by printing conditions and parameters [18,19]. For example, there are some major factors that interrupt the printing process such as comprising the applied voltages, printing distances (i.e., the distances between the nozzle tip and the substrate), and flow rates. On that basis, the controllability of the morphology of micro/nanostructures should be urgently increased. So far, some progress has been made; e.g., Sun Daoheng developed near-field direct writing technology where the printing stability is improved by reducing the printing height and printing in the jet stabilization stage [20]. Moreover, to control the fiber arrangement, an electric field is adopted to collect the charged jet back and forth between the flat electrostatic pole beam. Equally, a high-speed rotating drum is employed [21,22]. By adding magnetic particles to the solution and using an external magnetic field, the regulation of the deposition of fibers was also studied [23]. Furthermore, the printed structures significantly affect the stability of subsequent jets for the residual charge, and the printing stability can be affected [24]. In addition, the warping effect of the viscous solution can occur when the solution comes into contact with an unstable basal crimp [25].

On the other hand, some simulation studies have also made significant progress; i.e., in 2003, Yan et al. conducted a simulation with two-dimensional axisymmetric conditions by writing a design program and attained a geomorphology about the formation and atomization of a cone’s jet [26]. By solving the Navier–Stokes equation with electric stress and interfacial tension, the formation process of a Taylor cone, a liquid jet, and a droplet in the EHDA process was successfully simulated. The changes of the solution–air interface were tracked using the front tracking/finite volume method [27]. Qingxing Xu built a coaxial electrohydrodynamic atomization model supported by a CFD simulation, and it revealed that the velocity field development in a Taylor cone has a great influence on the distribution of the core and shell [28]. Yahya Kara presented a new method for preparing nano/microfibers by combining extrusion and melt blowing. The distribution of air velocity and the effect of wind speed on fiber diameter were simulated [29]. López-Herrera made an electrohydrodynamic axisymmetric model of coaxial electrosprays in 2020. They retained the electrical permittivities, immiscibilities, and conductivities of the solutions. Therefore, it existed in a region of the inner surface where the charge can be negative [30]. Shawn Dodds solved the Navier–Stokes equations with the finite element method for the stretching of a liquid drop between two surfaces for non-zero Reynolds numbers. The inertial-driven fluid was driven towards the surface with a higher contact angle, and wettability was established [31]. However, most current studies only consider the effects of voltage and the solution flow rate on jet morphology. Therefore, there are rarely discussions to improve the printing stability of low-viscosity functional solutions to fabricate uniform structures. Consequently, it is of great importance to conduct in-depth research to form aligned and uniform structures with smaller dimensions. Further, the shapes of the printed results must meet the demands of high-performance sensors and other microelectronics.

In this study, a high-viscosity solution was selected as the outer layer of a coaxial electrohydrodynamic jet (CE-jet), which was used to enhance the low-viscosity inner solution’s printing stability. The outer high-viscosity solution anchored the end of the coaxial jet to the moving substrate and dragged the jet, which provided a reference method to align the printed nanostructures. It was further used to improve the stability of low-viscosity ink and print high-resolution and customizable nanostructures. It utilized the combination of electrical and mechanical forces to reduce the printed structure’s size and improve the printing structure’s consistency. In addition, the drag force along the direction of movement reduced the jet trajectory instability caused by the repulsion of the jet and carried by the substrate with a residual charge. First, a force analysis model was built in terms of the moment of contact between the moving substrate and the end of the jet to analyze the different speed matches. Second, a finite element simulation model for coaxial E-jet printing was built based on the leaky dielectric model using COMSOL 5.2 (9.3.2, Flow Science, Santa Fe, NM, USA) to gain insights into the speed change and the speed distribution of the jet from generation to landing on the substrate. Furthermore, we printed an array of organic semiconductor indacenodithiophene copolymers with benzothiadiazole (IDT-BT) nanowires on a silicon dioxide substrate with optimized working conditions obtained from the simulation. Finally, we tested the device, which was fabricated via CE-jet printing with coaxial needles with inner diameters of 130 µm and 800 µm. Therefore, the CE-jet printing process can directly produce an arranged nanostructure with the help of the drag force supported by a movable substrate and a high-viscosity fluid.

## 2. Materials and Methods

### 2.1. Material Properties

The organic semiconductor polymer has been extensively applied in the field of Micro-Electro-Mechanical Systems (MEMSs). In this paper, the organic semiconductor polymer IDT-BT was selected as an inner solution for the simulation and experiment due to its excellent performance [32,33]. We purchased solid IDT-BT material (Nanjing Zhiyan Technology Co., Ltd, Nanjing, China) from a supplier and used o-dichlorobenzene (Aladdin, Shanghai, China) as a solvent. Moreover, it has a black color that can help to detect variations in jet flow during the printing process. Silicone oil (kinematic viscosity of 60,000 cst, Merck KGaA, Darmstadt, Germany/Shanghai, China) (Dow Corning Corporation, Midland, MI, USA), which has a high viscosity, was employed as an outer solution. Thus, a high-viscosity outer solution was used to improve the stability of the coaxial jet. It was fixed on the substrate by anchoring and dragging the jets and helped to thin the jet width [15]. The organic semiconductor polymer solution was synthesized in our laboratory (by setting the heating temperature to 90 °C and stirring for 12 h). The surface tension of the liquid was examined using a droplet shape analyzer (DSA100, Krüss GmbH, Hamburg, Germany). Table 1 provides a characterization of the materials used in the simulation and experiment throughout this work.

### 2.2. Experimental Setup

**Figure 1:**
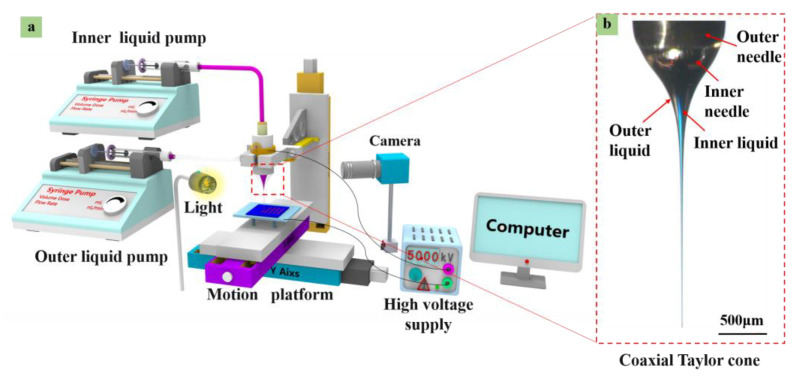
(**a**) Schematic diagram of the coaxial E-jet set up. (**b**) Partial enlarged drawing of coaxial jets.

Figure 1a presents an illustration of the coaxial printing setup. A coaxial needle comprised an inner needle with inner/outer widths of 130 µm/410 µm (New Objective, Inc., Woburn, MA, USA) and an outer needle with inner/outer diameters of 800 µm/1mm with silicon dioxide as the substrate. Two syringe pumps were used to provide the solutions accurately and independently. Furthermore, a direct current (DC) high-voltage power supply, a heating platform, a computer-controlled X-Y-Z movement platform, and a camera were used.

In the existing research, the hypotheses about the initial outline of the Taylor cone and the charge distribution on the cone-jet surface were proposed to speculate about the cone-jet formation process and its relevant features [34,35]. In 1969, Taylor described the conical shape formation of a jet as a hydrostatic balance between electrostatic forces and surface tension that can be matched [36].

**Figure 2:**
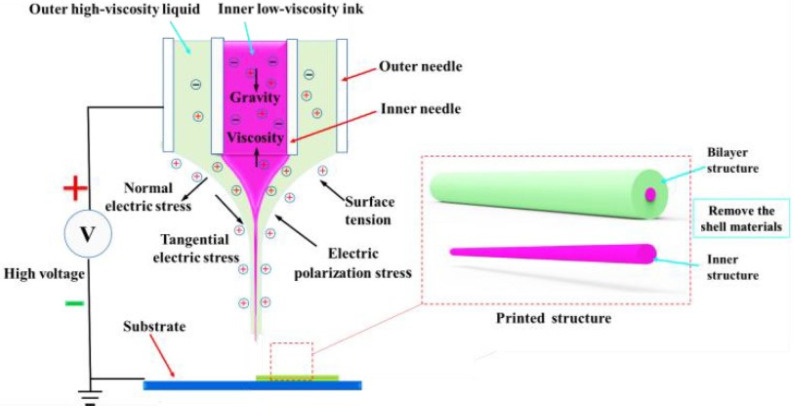
The distribution of active forces involved in the coaxial E-jet.

Figure 2 illustrates the forces exerted on the CE-jet contour. The forces in Figure 2 comprise the gravity of the solution, the electrostatic force generated from the external electric field, the Coulomb repulsion force, the viscoelastic force of the polymeric solution, and the surface tension between the air and solution. The electric field force can be decomposed into the normal field force and the tangential field force along the surface of the Taylor cone. The charges induced on the solution surface repelled each other and created shear stresses with the rise in the concentration of the electric field. The above-mentioned repulsive forces acted in a direction opposite to the surface tension, such as when the pendant jet was extended into a conical shape when the electric field force overcame the surface tension.

**Figure 3:**
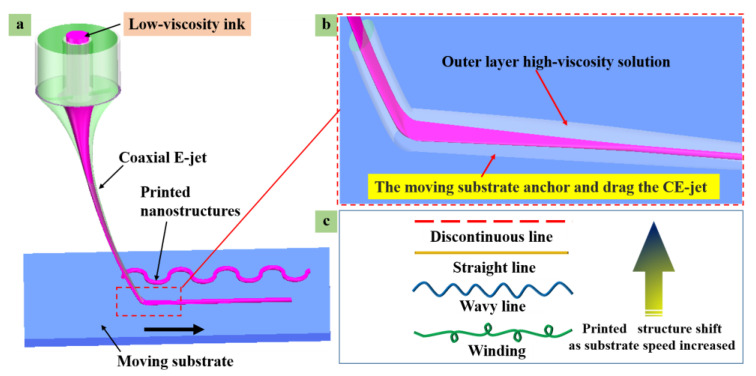
A schematic diagram of the forces involved in the moment of low contact between the coaxial E-jet and the moving substrate: (**a**) CE-jet is anchored and dragged by the substrate; (**b**) local diagram of end of jet; (**c**) schematic diagram of the printed result changing with the substrate’s moving speed.

When the jet reached the movable substrate, the end of the coaxial jet adhered to the substrate by its outer sphere’s high-viscosity solution, as depicted in Figure 3a,b. The electrostatic force expedited the jet flow, and the jet speed reached its maximum before buckling. The jet was subjected to upward resistance and slowed down after the substrate was obtained. In addition, the upward counterforce on the substrate caused the jet to whip after buckling. Thus, the deposition pattern variation bent in wavy, straight, and disconnected manners with the increase in the substrate movement, as shown in Figure 3c.

### 2.3. Modeling and Simulation Process

**Figure 4:**
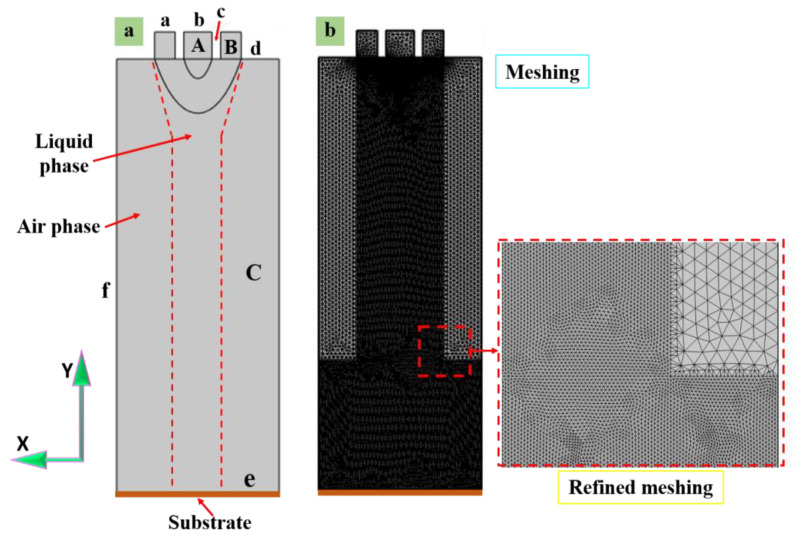
The simulation model for the CE-jet printing: (**a**) the geometric model, (**b**) the refined mesh for simulation.

The coaxial electric jet simulation comprised three phases (e.g., the outer solution phase, the inner solution phase, and the air phase). The potential electric field and fluid motion equation were combined with the intersecting interface charge coupling due to the application of an electric field. According to the theory of the leaky dielectric model, the solution inside the free charge in the electrostatic field under the action of migration gathered at the gas–solution interface. Considerable free charges gathered after the fluid in the cone of a shear “pull” and combined with the action of the mechanical force. An inverted cone profile can be formed to print a satisfactory jet much narrower than the nozzle diameter, as shown in Figure 4. A, B and C are the three main parameters of the geometric model loop. A is the generation of the inner needle loop and B is the generation of the outer needle loop. However, C is a loop of the needle body of the geometric model. Similarly, the boundary conditions of the geometric model of the phase field are denoted by symbols a through f.

The geometric model and the settlement of the boundary of the model are given in Table 2. An axisymmetric geometric model was established based on the actual experimental setup, as depicted in Figure 4a. The structure’s size was consistent with the actual size of the nozzle used in the experimental research. The inner and outer diameters of the inner nozzle were set to 130 µm and 470 µm, respectively. Likewise, the inner and outer diameters of the outer nozzle were set to 800 µm and 1 mm. The inner solution, the outer solution phase field, and air are represented as A, B, and C, respectively.

Table 2 summarizes the electrostatic field and flow field boundary conditions of the simulation model. Thus, φ expresses the electrostatic field potential and V is the voltage, varying in different locations. In addition, u and P represent the fluid velocity and pressure, respectively. Among them, “a” denotes the inlet of the inner layer liquid, which satisfies φ = V0 and u_i_ = Q_i_/A_i_, where V0 is the applied voltage on the coaxial needle. Moreover, Q_i_ is the flow rate of the inner layer liquid, and A_i_ is the effective flow area of the inner layer liquid. Thus, b is the inlet of the outer layer liquid, which satisfies φ = V0 and µ_o_ = Q_o_/A_o_, where Q_o_ is the flow rate of the outer layer liquid and A_o_ is the effective flow area of the outer layer liquid. Equally, c is the inner spray needle wall, which satisfies φ = V0 and u = 0. D is the outer spray needle wall, which refers to φ = V0 and u = 0. The symmetry axis of the 2D model refers to φ_r_ = 0 and u_r_ = 0. φr refers to the radial component of the electric potential, and u_r_ refers to the radial component of the flow ratio. Similarly, f is the boundary of the entire calculation domain, which satisfies φ = V and P = 0. Finally, g is the ground electrode and the outlet of the CE-jet, which satisfies φ = 0 and P = 0.

Table 2 lists the boundaries of the concrete numerical values, and the properties of the solution are listed in Table 1. The materials used in the simulation and experiment are the same. The moving mesh was adopted to represent the moving substrate. Likewise, the coupling was attributed to the effect of the substrate’s dragging on the jet shape. Where u is the fluid injection velocity, V0 is the applied voltage on the coaxial needle and V is the voltage, varying in different locations. Correspondingly, Q_i_ is the flow rate of the inner liquid, A_i_ is the cross-sectional area of the inner needle, and Q_o_ is the flow rate of the outer liquid. Finally, A_o_ is the difference in cross-sectional area between the inner and outer needles.

### 2.4. Numerical Approach

Fluid motion is obtained by solving the conservation of mass and the Navier–Stokes equations. According to the electrohydrodynamic theory, the CE-jet printing process is simulated and involves several aspects, including the fluid, electric field, and Cahn–Hilliard equation.

The Navier–Stokes terminologies are represented as follows in Equation (1):

(1) $$\rho \left(\frac{\partial u}{\partial t}+\left(\vec{u}\cdot \nabla \right)u\right)=-\nabla p+\mu \nabla u^{2}+{\vec{f}}_{e}+\rho \vec{g}+{\vec{f}}_{s}\;$$

where ρ $\mathrm{is}\;\mathrm{the}\;\mathrm{density}\;(\mathrm{kg}/m^{3})$, $\vec{u}$ is the velocity of the fluid, p is the fluid’s internal pressure (Pa), μ is the fluid’s viscosity coefficient, $\vec{g}$ is the gravitational acceleration, ${\vec{f}}_{e}$ is the electrostatic field force, and ${\vec{f}}_{s}$ is the surface tension at the gas–solution interface.

$\vec{f_{e}}$ is the electric field force. It is available through the Maxwell pressure tensor ($\sigma^{M}$):

(2) $$\vec{f_{e}}=\nabla \sigma^{M}=q_{v}\vec{E}-\frac{1}{2}E^{2}\nabla \varepsilon +\frac{1}{2}\nabla \cdot \left[{\rho \left(\frac{\partial \varepsilon }{\partial \rho }\right)}_{T}E^{2}\right]$$

where $q_{v}$ is the space charge density of two intersecting interfaces, E is electric field’s strength, ɛ is the fluid’s permittivity, and the subscript T is the temperature.

The first term in Equation (2) represents the Coulomb force, i.e., the result of the interaction between the charge on the interface of two phases and the applied electric field; its direction is consistent with the direction of the electro-strictive force. The second and third terms refer to the electric polarizing force and the restrictive electric force. The direction of the electric polarization force is perpendicular to the interface due to the existence of ∇ ε. The electro-strictive force is correlated with the change in fluid density and can be neglected, given the incompressibility of the fluid.

Maxwell tensor equations are used as an electrostatic mode in the phase field method [36]. The electric field is expressed in the form of electric potential. Hence, by applying Gauss’s law to a rectilinear electric medium, it can be reduced to Equation (3):

(3) $$\vec{f_{e}}=q_{v}\vec{E}-\frac{1}{2}E^{2}\nabla \varepsilon$$

An electrostatic field was connected between the printing nozzle and the grounding, and the Poisson equation was adopted to define the control of the potential according to the charge density, as given in Equation (4):

(4) $$\nabla \cdot \left(\varepsilon_{r}\varepsilon_{0}\nabla \varphi \right)=-q_{\nu }$$

(5) E=−∇φ

where $\varepsilon_{r}$ is the relative permittivity of the fluid, $\varepsilon_{0}$ is the dielectric constant of the vacuum, and φ is the electric field potential. An electrostatic field is connected between the printing nozzle and the grounding, and the Poisson equation defines the potential’s control according to the charge density, where $q_{v}$ is the space charge density. Since the electrostatic field is non-rotational, Equations (5) and (6) are calculated as follows:

(6) $$\nabla^{2}\varphi =0$$

where $\nabla^{2}$ denotes the Laplacian operator, defined as the divergence of the gradient. From the law of conservation of charge, as given in Equation (7), it yields:

(7) $$\frac{dq}{dt}=-\vec{n}\cdot \sigma (\nabla \varphi )$$

where $\frac{dq}{dt}$ denotes the charge change rate over time; σ represents the electrical conductivity; and $\vec{n}$ is normal to the gas–solution interface.

The VOF (Volume of Fluid) method has been extensively used in multi-media flow numerical simulation. The calculation becomes complicated or the physical quantity near the interface is not conserved due to the different interface reconstructions or re-initialization processes. Accordingly, the Cahn–Hilliard equation in COMSOL was employed in this study to control the interface motion [37] such that the above problems could be avoided. Equation (8) is expressed as follows:

(8) $$\left\{\begin{array}{c} \;\frac{\partial \varphi_{j}}{\partial t}+u\cdot \nabla \varphi_{j}=\nabla \cdot \frac{M_{0}}{\sum_{j}\;}\eta_{j}=\nabla \cdot \frac{\gamma \lambda }{\varepsilon^{2}}\nabla \psi \\ \eta_{j}=\frac{4\sum_{T}\;}{\theta }\sum_{j\neq k}\left(\frac{1}{\sum_{k}\;}\;\left(\partial_{j}F\left(\varphi \right)-\partial_{k}F\left(\varphi \right)\right)\right)-\frac{3}{4}\theta \sum_{j}\nabla^{2}\;\varphi_{j}\; \end{array}\right.\;j=A,B,C$$

where the function *F* is the Cahn–Hilliard potential, *j* is the phase field, and *θ* is a thickness parameter of the two-phase interface. Moreover, *M*_0_ is the mobility, which is taken as the function of the phase field variables, and the coefficient $\Sigma_{T}$ is defined as follows in Equations (9) and (10):

(9) $$\frac{3}{\Sigma_{T}}=\frac{1}{\Sigma_{1}}+\frac{1}{\Sigma_{2}}+\frac{1}{\Sigma_{3}}$$

(10) $$\phi_{A}+\phi_{B\;}+\phi_{C}=1$$

When the phase field variable ∅ reaches −1, 0, and 1, respectively, it represents the dielectric coefficient and air. *u* is the flow speed, and ∅ denotes the phase field parameter where the phase field variable is smoothest from −1 to 1. The surface tension (${\vec{f}}_{s}$) can be determined as follows in Equations (11) and (12) [38,39]:

(11) $${\vec{f}}_{s}=\xi \nabla \phi$$

where ξ represents the chemical potential factor (J/m^3^).

(12) $$\xi =\lambda \left[-\nabla^{2}\phi +\frac{\phi \left(\phi -1\right)}{\theta^{2}}\right]=\frac{\lambda }{\theta^{2}}\psi$$

*λ* expresses the mixed energy density, and ψ expresses the auxiliary variable of the phase field, as given in Equation (13):

(13) $$\tau =\mu \left(\nabla u+{\left(\nabla u\right)}^{T}\right)$$

where τ denotes the shear stress tensor and μ represents the dynamic viscosity of the fluid with the help of the diffusion interface representation.

## 3. Results and Discussion

### 3.1. CE-Jet Development Process

**Figure 5:**
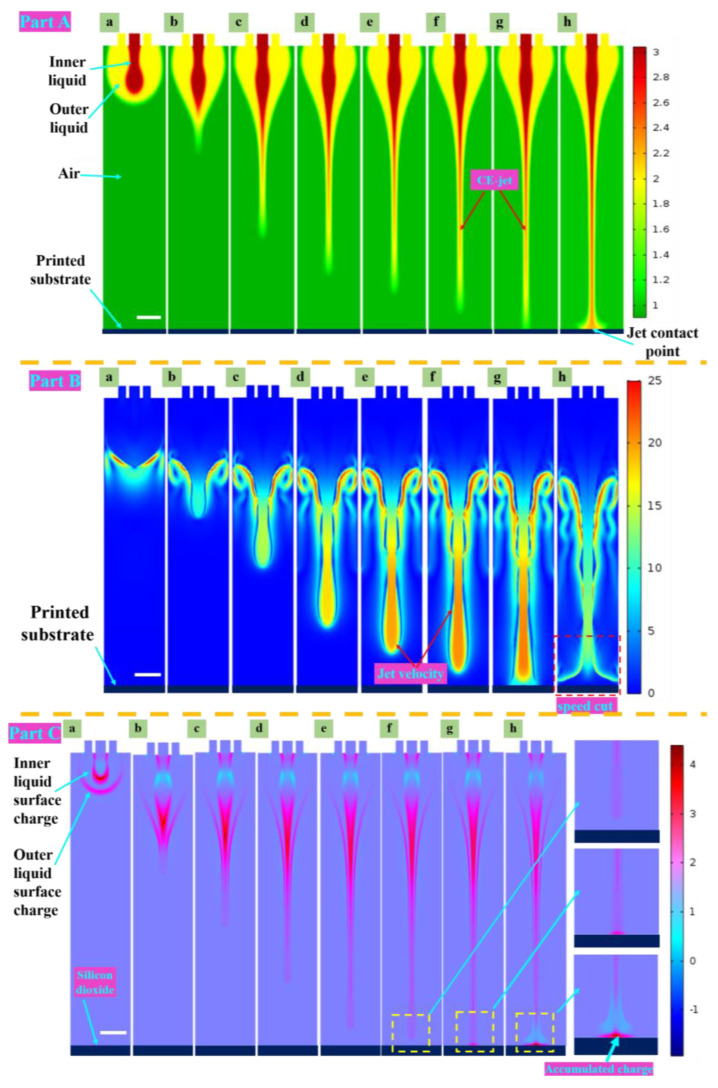
(**Part A**) The morphologies of ternary phase field simulation at different time steps of the coaxial jet printing formation process (a–h). (**Part B**) Schematic showing that the velocity distribution of the coaxial jet flies to the substrate at seven representative moments. (**Part C**) The charge density variation in the coaxial jet formation process. Scale bar = 500 µm.

The electric field served as a crucial factor for the CE-jet printing technique. Figure 5 presents the geomorphologies of the simulated CE-jet at different time intervals under the applied voltage of 5.5 kV. However, other parameters (e.g., the distance between the spray needle and the substrate, and the flow rates of the inner and outer solutions) remained unchanged. The core solution region marked as red was defined as phase A, the outer solution marked as blue was defined as phase B, and the air domain marked as dark blue was defined as phase C. As depicted in Figure 2, the forces in the CE-jet formation process primarily comprised the viscous force, surface tension, and gravity, as well as the electric field force.

The solution droplets in the meniscus shape were formed at the nozzle outlet under the viscous force of surface tension and gravity when no electric field force was applied, as shown in Figure 5a. According to Melcher’s leaky dielectric model and the theory proposed by Taylor [40], a solution will have a free charge when an electric field is applied to the track interface beside the needle tip. With the application of the electric field, the charged particles in the solution will move under the electric field force. The particles with an identical polar charge to the electrode will be repelled away from the electrode and then gathered at the tip of the Taylor cone. The electric field force at the tip will be too small to overcome the tip’s viscous force and surface tension under a lower voltage, as illustrated in Figure 5b. The electric field force tends to overcome the surface tension to form a jet with a rise in the applied voltage. Lastow et al. reported the configuration of charge distribution [41] until the electrical shearing force is adequate to overwhelm the surface tension, as the charge gathers to a certain extent. On that basis, a CE-jet tends to be formed and the flight towards the substrate is accelerated.

In this study, a positive voltage was applied at the injection needle as depicted in Figure 5 (see Part C). Therefore, the surface of the jet collected positive charges. When the jet printed on the insulation substrate (silicon oxide) (see Figure 5, Part C (h)), the charges carried by the jet accumulated and were hard to transfer away. The substrate carried out the charges of the same polarity as the jet when a certain degree of charge accumulated. Thus, it caused repulsion to the subsequent jet flight motion and affected the stability of the printing process.

The electric field force is the key to the formation of a stable jet. The jet is accelerated to the substrate until it is connected with the negative plate (substrate) under the action of the electric field force. The instantaneous jet speed reaches its maximum value before reaching the substrate. Figure 5 (Part B) presents the distribution of jet speed under the electric field force. The instantaneous jet speed reached its maximum value before reaching the substrate. When the jet descended to the base (see Figure 5, Part B(h)), the jet speed declined instantaneously due to the collision between the jet and the substrate. Therefore, the charge it carried repelled the residual charge of the printed structures on the substrate. Lastly, curls with irregular lines were formed.

Therefore, we used the high-viscosity outer liquid of the coaxial jet to improve the stability of the jet flight process. The inner jet exploited the viscous force of the outer high-viscosity solution, and the printing stability of the inner low-viscosity solution was increased. Moreover, the high-viscosity outer solution contributed to fixing on the substrates to form uniform structures. The CE-jet was anchored after the jet landed on the substrate, and the moving substrate was adopted to drag the jet. Similar to the wind competition, the aim was to control the stability of the jet flight. Furthermore, the relative sliding of the inner and outer layers of the solution was adopted to further shear and refine the printing of the inner structure.

**Figure 6:**
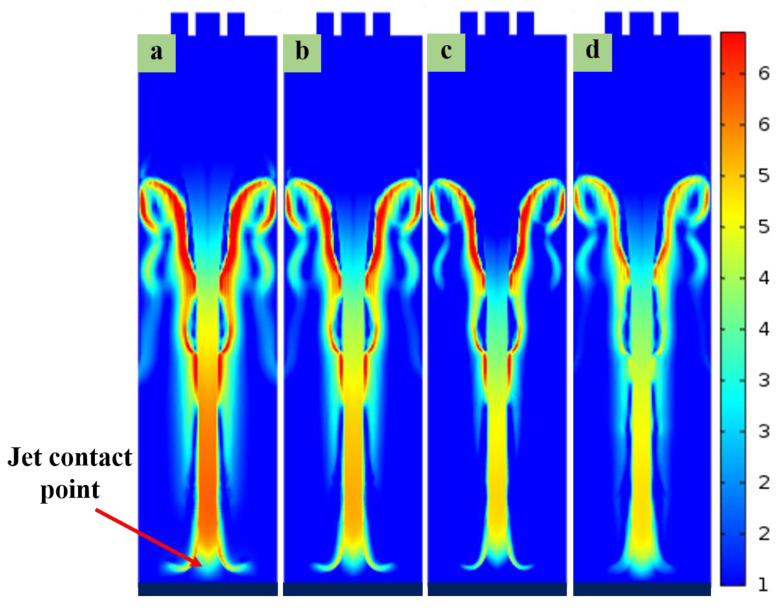
Schematic showing the specific morphologies of the CE-jet velocity (*V*_J_) before it contacts the substrate at different applied DC voltages: (**a**) 7.0 kV, (**b**) 6.5 kV, (**c**) 6 kV, and (**d**) 5.5 kV.

The Coulomb repulsion increases with the charge density due to the rise in applied voltage. Therefore, when the applied voltage is increased, the electric field force on the jet increases and the jet has more significant speed during the flight. Accordingly, the speed at the end of the jet declined with the reduction in the applied voltage at the moment before the flight touched the substrate, as shown in Figure 6. The voltage applied between the injection needle and the substrate reached (a) 7.0 kV, (b) 6.5 kV, (c) 6 kV, and (d) 5.5 kV. Furthermore, the velocity distribution as the end of the CE-jet reached the substrate was simulated.

### 3.2. Experimental Study of Effect of Substrate’s Moving Speed on Printed Structures

The specific speed has been characterized as a critical parameter in the electrojet printing process [42]. An impact will be generated between the grafting collection and the jets when the accelerated jets are deposited on the collection. As a result, curvature instability of the threads will be caused since the substrate’s moving speed is lower than the jet speed and the jet will be bent immediately after reaching the substrate [43,44]. The above-mentioned instability causes the deposition patterns from the sinusoidal line to twist, fold, and spiral.

#### 3.2.1. Substrate’s Moving Speed Affected Printed Structure Pattern

**Figure 7:**
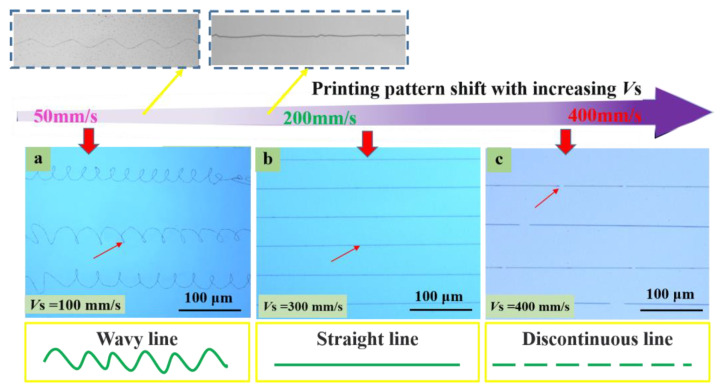
The pattern of the printed structure changed with the speed of substrate movement. (**a**) A bending curve prepared when the jet speed was greater than the substrate (*V*_J_ > *V*_S_). (**b**) A uniform smooth line prepared when the substrate dragged the jet at an appropriate velocity (*V*_J_ < *V*_S_). (**c**) A break line prepared when the substrate transition dragged the jet (*V*_J_ < < *V*_S_).

**Figure 8:**
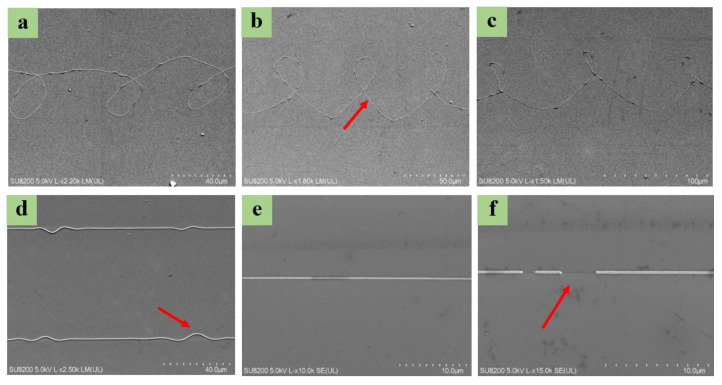
Patterning with applied voltage and other parameters fixed while the substrate speed increased. (**a**–**f**) Structures printed by altering substrate speed during continuous CE-jet printing with coil shape, wavy pattern, straight line, and discontinuous line. Switching between different lines on the same substrate was controlled by the substrate movement speed.

Figure 7 demonstrates the corresponding experimental results, which verified the influence of the moving speed on the shapes of structures printed with CE-jet printing. The inner fluid was an IDT-B organic semiconductor polymer, and the outer fluid was a silicone oil with a viscosity of 60,000 centistokes (CST), as determined in the simulation. The internal and external fluid flow rates were adjusted to 150 nL/min and 3 µL/min, respectively. The sizes of the injection needles were 130 µm and 800 µm. The print height was kept at 2.0 mm. For the experiments, Figure 8 shows the situation when the speed of the moving substrate was increased (20 mm/s, 50 mm/s, 100 mm/s, 200 mm/s, 300 mm/s, and 420 mm/s) while printing various structures.

Mainly, the velocity of the CE-jet *(V*_J_) (*V*_J_ is the velocity of the substrate) is greater than the moveable substrate speed (*V_S_)* (*V_S_* is the velocity of the substrate). The relationship between two separate parameters of velocities is discussed as *V*_J_ > *V_S_*. After the jet is deposited on the substrate, it receives the upward supporting reaction force, and then the jet is compressed. This results in the bending and buckling phenomenon. When the buckling reaches a certain degree, the jet will twist in space and vibrate erratically or rotate about the nozzle axis. In this case, a curl, zigzag, or other pattern will appear along the direction of the substrate. Moreover, the amplitude and wavelength of the pattern depends on the velocity of the substrate, as depicted in Figure 8a–c.

The material properties of the substrate (*V_S_*) and the CE-jet liquid jet velocity (*V*_J_) are distinctive parameters for approximating the jet extension around the axis of deposition at the nozzle interface. Therefore, if the substrate to the jet stream tension moves with an equal movement of the substrate, then the cone jet cannot form a catenary shape. However, its amplitude is smaller at certain points. This is generally the point of demarcation between the curl pattern and the straight pattern, caused by bending and buckling as shown in Figure 8d. If the jet speed is less than the substrate velocity (*V*_J_ < *V_S_*), then jet deposition on the substrate and substrate adhesion on the end of the traction are observed during the printing process. Equally, the catenary form along the substrate movement direction and the jet deposition in a straight line were also investigated within the horizontal positions of the printed lines, as shown in Figure 8b. When the jet speed is much smaller than the substrate speed, such as *V*_J_ < < *V_S_*, then the inner solution of the coaxial jet will be pulled apart due to the high-speed traction of the substrate. Moreover, it is pulled off by falling on the substrate to form discontinuous broken lines, which are shown in Figure 8f.

#### 3.2.2. Influence of Substrate’s Moving Speed on Printed Structures’ Diameters

**Figure 9:**
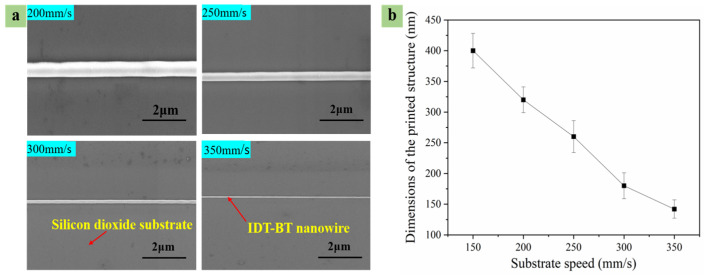
The fiber diameters measured for four substrate speed values. (**a**) Fitting of the diameter response to the substrate speed. (**b**) Effect of substrate’s moving speed on line width.

In a specific speed range (*V*_J_ < *V_S_*), with a rise in the substrate’s moving speed, the volume of solution falling on the substrate was reduced per unit time, and dragging the jet by the moving substrate refined the jet. The moving speed of the substrate was (e.g., 200 mm/s, 250 mm/s, 300 mm/s, and 350 mm/s states) obtained while printing polymer micro-nanostructures of different widths, as shown in Figure 9a. Thus, the effect of the substrate’s moving speed on line width is shown in Figure 9b.

**Figure 10:**
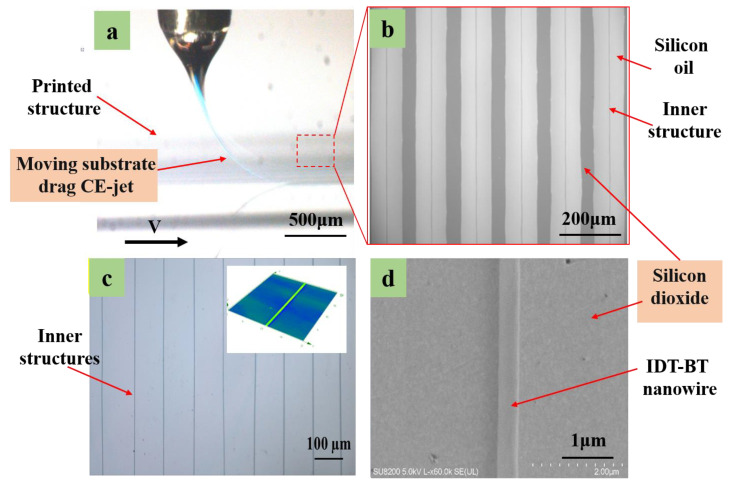
(**a**) Experimental picture of coaxial electric jet printing on moving substrate. (**b**) Array of IDT-BT and silicone oil double-decker line structures. (**c**) Array of IDT-BT nanowire after removing the outer layer solution. The illustration picture is a 3D profile of nanowire. (**d**) SEM images of printed IDT-BT nanowire.

Based on the above optimized experimental parameters, submicron IDT-BT line structures were printed under an internal flow rate of 4.0 × 10^−16^ m^3^/s using 8 mg/mL IDT-BT and an external flow rate of 2.6 × 10^−11^ m^3^/s. A substrate movement rate of 300 mm/s was applied under the conditions of using silicone oil as the outer auxiliary solution, an inner needle with an inner diameter of 130 μm and an outer diameter of 420 μm, and outer needle with an inner diameter of 0.8 mm and an outer diameter of 1 mm. The printing height of the spray needle from the substrate was maintained at 3 mm. The experimental image of coaxial electric jet printing on the moving substrate was captured using a view camera indicating the inner solution in the coaxial jet, i.e., the blue semiconductor solution jet, as shown in Figure 10a. Moreover, the coaxial jet formed a catenary state under the drag of the moving substrate. Figure 10b presents an array of double-layer composite structures printed on a silicon dioxide wafer substrate, suggesting that the inner functional material was well wrapped in the outer solution and then deposited on the substrate surface. The inner layer structure was not damaged after the exterior auxiliary solution was removed. It was firmly combined with the substrate’s upper surface. The printed array was constructed in an isopropyl alcohol solution to soak for 30 min and then dried. Subsequently, the remaining inner structure of functional materials (IDT-BT) remained in the array line without deformation, as shown in Figure 10c. The printed structures (consisting of the inner functional material) were smooth, and the line width was uniform. Scanning electron microscopy (SEM) images are presented in Figure 10d. The smallest size of these structures reached ~100 nm.

#### 3.2.3. Electrical Property Performance of Printed Structures

**Figure 11:**
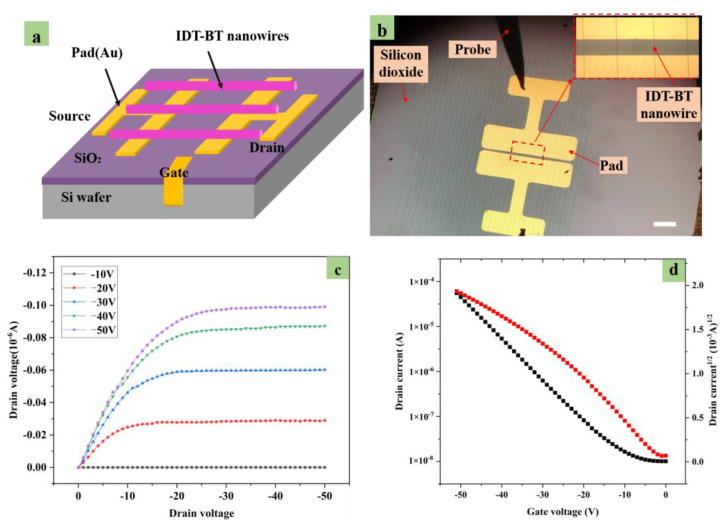
(**a**) Schematic of an OFET from an array of IDT-BT fibers. (**b**) Optical microscope image of the OFET sample with an array of IDT-BT fibers that span Au (150 nm thick) electrodes forming the device. The right inset represents a partial magnification micrograph of OFET. The silicon wafer forms a bottom gate for the device. Scale bar = 100 µm. (**c**) Output curve of OFET from array’s IDT-BT nanowires. (**d**) Transfer characteristic of the OFET worked at a constant drain of −50 V. Scale bar = 100 µm.

Figure 11a,b illustrate a device that was constructed on an array of IDT-BT fibers, and its electrical characteristics are depicted. The distance between two parallel electrodes (L) was 50 µm (see Figure 11c) and the diameter of the fibers was nearly 150 nm, along with the width (L = n × 150). The channel current could be expressed as *I*_DS_ = (*W*/2*L*) *C_i_ µ*_FE_ (*V*_G_ − *V*_T_) ^2^, where *µ*_FE_ represents field-effect mobility, *C*_i_ denotes capacitance per unit area of the insulation layer (in this study, Si/SiO_2_ (300 nm); C = 11 nF.cm^−1^), and *L* expresses the length of the channel. Similarly, *W* is the width of the channel, *V*_T_ is the threshold voltage, *V*_G_ denotes the gate source bias, *V*_DS_ expresses the drain source bias, and *I*_DS_ is the source-drain current. Figure 11d presents the output characteristic curve from the field-effect transistor (*I*_D_−*V*_DS_), and the *V*_T_ is −8V. The field-effect mobility was estimated to be 0.0688 cm^2^·V^−1^·s^−1^. Furthermore, the array fibers’ transistor characteristics were measured in an ambient atmosphere using an Agilent 4200C semiconductor parameter analyzer (Hercules, CA, USA) that was connected to a Karl Suss (PM5) manual probe station.

## 4. Conclusions

Combinations of electrical and mechanical forces, rather than purely electric fields as in conventional (NFES) ink-jet printing, were adopted to drive low-viscosity ink to determine the uniformity of string patterns. Moreover, a mobile structure was adopted to anchor and drag the jet landing on its surface for shear refinement and to reduce the size of the printed structure. The consistency of the printed structures was enhanced. The jet trajectory instability arising from the structure carrying a residual charge to the jet repulsion was reduced. The change in the jet speed during jet flight and the effect of various working voltages on jet speed were simulated in this study. The mechanical drawing force controlled by the substrate speed took on critical significance for the improvements over NFES techniques in the resolution, positioning, and alignment of micro/nanofibers. Array structures were prepared by matching the moving speed of the structure to the jet speed under different voltages. The experimental results were verified based on the simulation. Lastly, a wide variety of resolutions and morphologies of micro/nanostructures were fabricated effectively by adjusting the substrate speed. Furthermore, the OFET was prepared by adapting the appropriate printing platform moving speed. The speed was adjusted to be suitable for printing the pre-prepared structures with the use of electrodes. The method proposed in this study can provide a reference for increasing the printing stability of low-viscosity solutions.

## Figures and Tables

**Table 1:** The properties of IDT-BT liquid and silicone oil.

Character	Dynamic Viscosity µ (Pa.s)	Density ρ (kg m^−3^)	Relative Permittivity ε	Surface Tension Coefficient σ (mN.m^−1^)
Silicone oil	58.56	976	2.77	21
IDT-BT liquid	12	16	22.5	12.5

**Table 2:** The boundary conditions of the simulation model.

Boundary	Electrostatic Field	Hydrodynamic Field
a: Inner needle inlet	φ = V_0_	u_i_ = Q_i_/A_i_
b: Outer needle inlet	φ = V_0_	u_o_ = Q_o_/A_o_
c: Wall of inner needle	φ = V_0_	u = 0
d: Wall of outer needle	φ = V_0_	u = 0
e: Boundary of computational domain	φ = V	P = 0
f: Axisymmetric	φr = 0	u_r_ = 0
g: Outlet	φ = 0	P = 0

## Data Availability

Not applicable.
